# The Role of Plasmacytoid Dendritic Cells in Cancers

**DOI:** 10.3389/fimmu.2021.749190

**Published:** 2021-10-19

**Authors:** Binhui Zhou, Toby Lawrence, Yinming Liang

**Affiliations:** ^1^ Laboratory of Mouse Genetics, Institute of Psychiatry and Neuroscience, Xinxiang Medical University, Henan, China; ^2^ Laboratory of Genetic Regulators in the Immune System, Henan Collaborative Innovation Center of Molecular Diagnosis and Laboratory Medicine, Xinxiang Medical University, Henan, China; ^3^ Henan Key Laboratory of Immunology and Targeted Therapy, School of Laboratory Medicine, Xinxiang Medical University, Henan, China; ^4^ Centre for Inflammation Biology and Cancer Immunology, King’s College London, London, United Kingdom

**Keywords:** plasmacytoid dendritic cells, malignancy, regulatory T cells, type I interferon, immunosuppression

## Abstract

Plasmacytoid dendritic cells (pDCs) are a special subtype of dendritic cells with the morphology of plasma cells. pDCs produce massive amounts of type I interferon (IFN-I), which was originally found to play an extremely pivotal role in antiviral immunity. Interestingly, accumulated evidence indicates that pDCs can also play an important role in tumorigenesis. In the human body, most of the IFN-α is secreted by activated pDCs mediated by toll-like receptor (TLR) stimulation. In many types of cancer, tumors are infiltrated by a large number of pDCs, however, these pDCs exhibit no response to TLR stimulation, and reduced or absent IFN-α production. In addition, tumor-infiltrating pDCs promote recruitment of regulatory T cells (Tregs) into the tumor microenvironment, leading to immunosuppression and promoting tumor growth. In this review, we discuss recent insights into the development of pDCs and their roles in a variety of malignancies, with special emphasis on the basic mechanisms.

## Introduction

Plasmacytoid dendritic cells (pDCs) are a unique subgroup of dendritic cells (DCs) with plasma cell morphology and have been extensively studied in recent years. The main function of pDCs is the production of IFN-I following recognition of viruses or nucleic acids through TLR7 and TLR9 (1, 2). Therefore, pDCs play a pivotal role in antiviral immunity. Previous studies have shown that DCs are critical in mounting effective immune responses to cancer (3–6). However, pDCs have received less attention in tumor immunity than other DC subgroups. In fact, similar to cDCs, pDCs link the innate and adaptive immune responses by regulating the biological function of lymphocytes, myeloid DCs and NK cells through producing two kinds of pro-inflammatory cytokines including tumor necrosis factor (TNF)-α and interleukin (IL)-6 (1, 7), and play an important role in cancer immunity.

pDCs are continuously produced from hematopoietic stem cells in the bone marrow (BM) and emerge as mature cells into the periphery (8). Under steady state conditions, the pDC precursor cells in the bone marrow enters the blood circulation, and then enters the secondary lymphatic tissue through the lymphatic circulation. In addition, a small amount of pDCs are also observed in the peripheral tissues such as liver, lung and gut, while they are believed to be absent in the skin (9). Interestingly, previous publications reported that pDCs infiltrate various types of solid tumors, including head and neck, liver, breast, colorectal, ovary, stomach, lung and skin cancers (10–17). Depending on the microenvironment and the type of stimulus, pDCs are capable of exerting either immunogenic or tolerogenic functions (18–20). In this review, we summarize current knowledge about the role of pDCs in different malignancies.

## PDCS Development and Identification

The development of pDCs depends on multiple factors including Flt3 ligand (Flt3L) (21), transcription factor Spi-B (22, 23), and the basic helix-loop-helix protein E2-2 (24, 25). Among them, Flt3L together with Flt3 activate transcription factor E2-2 in a STAT3-dependent manner to control the expression level of transcription factors necessary for the development and function of pDC (18, 26). Spi-B regulates human plasmacytoid dendritic cell survival through direct induction of the antiapoptotic gene BCL2-A1 (27), and plays a key role in pDC differentiation, whereas BCL11A activation is shown to direct CDP commitment to pDC lineage and regulate the transcriptional level of E2-2, Id2, Id3 and Mtg16 through a positive feedback loop (18, 22, 28). In addition, transcription factor E2-2 also plays an essential and specific regulator in pDC development (29, 30). By using single-cell sequencing, Ginhoux’s team showed that pDCs developed from a Ly6D^high^CD2^high^ lymphoid progenitor cell in the bone marrow and differentiated independently of the myeloid cDC lineage (31).

Concerning their identification, human pDCs express CD4, blood-derived dendritic cell antigen 2 (BDCA2, also termed CD303), CD123 (IL-3R), HLA-DR, ILT3 and ILT7 on the surface, and Toll-like receptor (TLR)7 and TLR9 within endosomal compartments (7, 30, 32), but lack most of the lineage surface markers for T, B, natural killer (NK) cells and monocytes (33, 34). And in mice, pDCs not only express B220 (CD45R), CD11c and Ly6C (35), but also express a variety of factors that modulate the function of pDCs, such as Siglec-H, Bst-2, Pdc-Trem and Ly49Q (1, 36).

## Role of PDCS in Cancers

### PDCS and Melanoma

**Figure 1:**
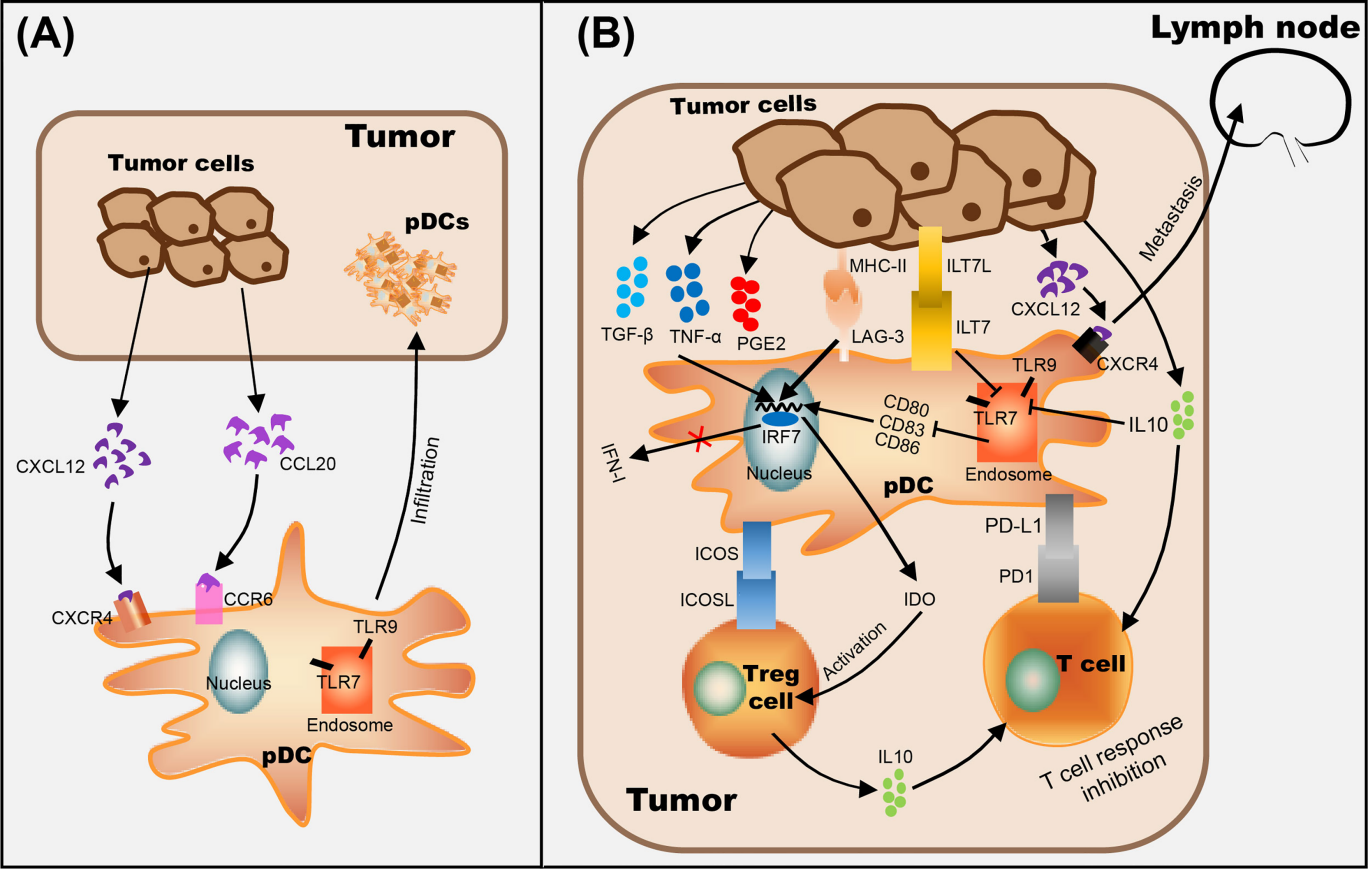
The role of pDCs in tumor progression. **(A)** pDC recognizes CCL20 secreted by tumor cells through CCR6 on the cell membrane and makes it migrate to the tumor site. **(B)** Mechanisms of tumor-infiltrated pDC on immunosuppression include recruitment of immature pDCs lacking the expression of costimulatory molecules (through CCR6/CCL20 pathway), suppression of type I IFN secretion by pDCs (by ILT7L-ILT7 interaction or immunosuppressive cytokines secreted by tumor cells, such as IL-10), alternate pDC activation (through the interaction between LAG-3^+^ pDC and MHC II^+^ tumor cells), and/or promote pDC tolerance by activating Tregs (through ICOSL/ICOS interaction and IDO production) and enhancing the expression level of anti-inflammatory cytokine IL-10. In addition, the up-regulation of CXCR4 on the surface of pDC and the promotion of CXCL12 secretion by tumor cells are positively correlated with lymph node metastasis of tumor cells.

Functional studies of pDCs in cancer have mostly focused on mouse models of melanoma. Despite the fact cutaneous melanoma is a highly immunogenic solid tumor, the occurrence and development of melanoma is related to its ability to escape immunosurveillance (7). Previous studies have shown that circulating pDC levels were decreased in blood of melanoma patients (37), however, pDCs were increased in primary tumors and tumor-draining lymph nodes, and pDC infiltration was associated with poor prognosis and early relapse (38, 39). In addition, melanoma cells were shown to recruit pDCs into the tumor microenvironment *via* stromal-derived factor-1 (SDF-1, also named CXCL12) (40). Moreover, IL-3 up-regulates the expression of chemokine receptor 6 (CCR6) in pDCs, as another mechanism for pDCs recruitment into the tumor microenvironment in melanoma, through CCR6/CCL20 (chemokine ligand 20) activation (41) (**Figure 1A**). Aspord et al. showed that pDCs in melanoma triggered IL-5-/IL-13-producing CD4 type 2 T helper (Th2) cells and IL-10-producing Tregs through the expression of OX40L and ICOSL, the secretion of Th2 cytokines leading to melanoma progression (39). Moreover, the expression level of MxA (a IFN-α inducible protein) in primary cutaneous melanomas was drastically inhibited in the majority of the cases (42) and the poor IFN-α production by pDCs has been associated with melanoma growth (40, 42, 43). Interestingly, Aspord and co-workers additionally demonstrated that the development of melanoma was strongly inhibited by imiquimod treatment (a TLR7 agonist) using an innovative melanoma-bearing humanized mouse model (44). They found pDCs in tumor site were mobilized and their cytotoxic functions were increased, in addition, the expression levels of type I IFN (IFN-α) response genes were up-regulated, thereby inhibiting melanoma growth (44). Another study suggested that CpG B-type oligodeoxynucleotide (ODN) PF-3512676 activates pDCs in the sentinel lymph nodes of melanoma patients through the TLR pathway, prompting pDCs to release IFN-α, thereby enhances antitumor immunity (45). Furthermore, *in vitro* experiments have shown that the expression of cytotoxic molecule TRAIL was induced on pDCs by virus, imiquimod or IFN-α stimulation, and can be used to effectively lyse melanoma cells (7, 46, 47) (**Table 1**).

**Table 1:** Therapeutic approaches for various cancers through pDCs.

Type of cancers	Role of pDCs	Therapeutic approach	References
Melanoma	Limit IFN-α secretion, recruit Tregs and enhance immunosuppression.	TLR9-agonist	(45, 48, 49)
		TLR7-agonist	(46, 50)
		TLR-4 ligand	(51)
		Pim-3-targeting bifunctional shRNA	(52)
		IFN-α therapy	(53)
		IFN-α therapy + checkpoint inhibitor	(54)
		pDC-based vaccination	(55–57)
Lung cancer	Induce immunosuppression and promote the proliferation of lung cancer cells.	TLR4-agonist	(58)
Gastric cancer	Promote the differentiation of naive CD4^+^ T cells into Tregs and facilitate tumor immune escape.	TLR3 agonist	(59)
Breast cancer	Contribute to the immune escape of breast cancer cells and promote tumor growth.	–	–
Liver cancer	Promote Tregs to produce IL-10, thereby inhibit T cell responses and assist immunosuppression and tumor progression.	–	–
Squamous cell carcinoma	Limit IFN-α secretion and promote tumor progression.	CD317 antibody	(60)
Leukemia	Recruit Tregs into CMML.	CD123-targeted therapy	(61)
Ovarian cancer	Limit IFN-α secretion recruit Tregs and enhance immunosuppression	Prophylactic vaccines	(62)

Numerous studies have shown that the infiltration of a large number of pDCs is related to immunosuppression in the tumor microenvironment (12, 63–66) (**Figure 1B**). Evidence suggested that the interaction between LAG-3 and MHC-II induced TLR-independent activation of pDCs with enhanced IL-6 and limited IFN-α secretion, induced the production of CCL2 in monocytes, and generated Tregs from allogenic CD4^+^ CD25^-^ T cells, which ultimately leads to immunosuppression in tumors (67, 68). On the other hand, ILT7L was reported to down-regulate the expression level of IFN-α through its interaction with ILT7 receptors, and IDO (indoleamine 2,3-dioxygenase) released by pDCs strongly promotes the activation of Tregs, which leads to anergy, eventually helping tumor cells escape immune surveillance (69, 70). In addition, *in vitro* experiments showed that activated pDCs up-regulate the expression levels of MHC class I and class II molecules and CD95 on melanoma cells (71), and researchers speculate that tumor cells are more easily recognized by CTL *in vivo* (72). However, even if pDCs were activated in tumors, only weak and cytoplasmic expression of CD95 was detected on melanoma cells, suggesting that the progressive loss of CD95 in tumor cells as a possible mechanism of tumor escape (71). Melanoma cells have also acquired mechanisms to subvert the immune-stimulatory functions of pDCs, such as secrete immunosuppressive cytokines, including IL-10, TGF-β and PGE2, to suppress the expression level of TLR7/9 and IRF7, resulting in pDCs producing only a small amount of I-IFN (43, 73). Moreover, Wnt5a was strongly expressed in melanoma cells which suppressed the activation and IFN-α production of pDCs stimulated by CpG oligodeoxynucleotide, thus weakening the anti-tumor effects of CpG (73).

In recent years, different approaches have emerged for the treatment of melanoma that affect pDC functions. For example, ponophosphoryl lipid A (MPLA), a toll-like receptor 4 ligand, exhibits the capability to enhance anti-PD-L1 antibody-mediated anti-cancer immunity by activating pDC to produce IFN-α (51). Furthermore, the ssRNA-Pim-3-shRNA dual-function therapy established by Liu’s group not only enhanced the activation and IFN-α secretion of pDCs, promoted the apoptosis and inhibited the proliferation of melanoma cells, but also enhanced the activation of CD8^+^ T cells and NK cells and simultaneously reduced the proportion of Tregs and myeloid-derived suppressor cells (MDSCs), and ultimately reversed the tumor immunosuppressive microenvironment (52). Targeted delivery of IFN-α into the tumor site enhance the local immune response and the benefit of the checkpoint inhibition (53). Interestingly, the combination of intratumoral injection of IFN-α and anti-PD-1 immunotherapy (Clinicaltrials.gov research identifier: NCT02339324) suppressed PD-L1-mediated escape (43, 54). In addition, subcutaneous injection of TLR9-activating oligodeoxynucleotide PF-3512676 enhanced activation of pDCs and cytotoxicity of NK cells (48). Hofmann et al. also injected PF-3512676 in cutaneous or subcutaneous melanoma metastasis of 5 patients with melanoma in a phase I study, and observed local tumor regression (49). Furthermore, the combined topical use of imiquimod and monobenzone caused local regression of cutaneous metastases in 52% of 21 melanoma patients (stage III-IV) in a phase II study (50). On the other hand, by activating autologous pDCs and simultaneously loading with melanoma-associated peptides, and then injecting them into the lymph nodes, induced a systemic IFN-I response and activated NK cells (55). Other studies support the development of a pDC-based vaccine (HLA-A^*^0201^+^ pDCs) to produce tumor-specific T cells for adoptive cellular immunotherapy in melanoma patients (56, 57) (**Table 1**).

### PDCS and Lung Cancer

Lung cancer has a very high morbidity and mortality rate in the smoking population (74). According to the morphology of cancer cells, lung cancer can be divided into four subtypes, including small cell carcinoma, adenocarcinoma, squamous cell carcinoma, and large cell carcinoma (75). Like other types of cancer, lung cancer is also accompanied by a drastic accumulation of pDCs (63, 76). Previous research has shown that pDCs are robustly increased in the peripheral blood of non-small cell lung carcinoma (NSCLC), and the degree of pDC accumulation is related to the clinical grade of disease (76). Another study also observed that tumor-infiltrating pDCs (TIpDCs) were significantly increased in lung tumor masses compared to healthy tissues, these pDCs expressed higher levels of CD33 and PD-L1, associated with reduced cytotoxic activity towards tumor cells and in fact promoting their proliferation (63). Moreover, TIpDCs produced higher levels of IL-1α, which promotes angiogenesis and enhances the invasiveness of cancer cells, thereby promoting the progression of lung cancer (63). On the other hand, Perrot et al. reported that the expression levels of the activation markers CD80, CD83, CD86, or CD208/DC-LAMP on pDCs infiltrating NSCLC, were completely suppressed and only partial upregulation of CD86 was detected after TLR7 activation. In addition, even after TLR9 stimulation, only very weak T cell proliferation and IFN-α secretion was induced by TIpDCs. Therefore, the abnormal differentiation of TIpDCs seems to be an additional factor contributing to tumor immune escape (77).

It is worth noting that in previous studies, CpG-oligodeoxynucleotides can stimulate the activation of pDCs and induce anti-tumor immunity in a mouse model of melanoma (73). However, in the lung cancer microenvironment, the anti-tumor effect of CpG-oligodeoxynucleotides is ineffective, and the accumulation of pDCs promotes the tumor infiltration of Tregs and immature myeloid dendritic cells (mDCs), thereby inducing immunosuppression and promoting the proliferation of lung cancer cells (16). These studies show that the activity of pDCs is regulated by the tumor microenvironment, and the role of pDCs is multifaceted in different types of tumors.

Interestingly, numerous studies have reported the anti-tumor effects of LPS (78–82). In a mouse model of melanoma-induced metastatic lung cancer, Rega et al. showed that the administration of low-dose LPS caused immunosuppression, which was associated with the infiltration of pDCs, Tregs, MDSCs and CD8^+^ Tregs, while the growth inhibition of lung tumor caused by large dose of LPS was associated with the massive infiltration of pDCs, as well as Th1 and Th17 polarization (58).

### PDCS and Gastric Cancer

Gastric cancer (GC) is one of the five most common cancers worldwide as well as the third biggest cause of cancer-related mortality (83). Previous studies have shown that pDCs play a crucial role in GC (66). Although the population of pDCs in the peripheral blood of GC patients is significantly elevated, the plasma concentration of IFN-α was significantly decreased (66, 84). On this basis, circulating pDCs showed a positive correlation with advanced stages and lymph node metastasis in gastric cancer (84). In addition, the accumulation of pDCs in peripheral blood and tumor tissues predicted poor clinical outcome in GC patients (85).

Previous studies have shown that gastric microbiota dysbiosis and immune system dysfunction are critical factors for the occurrence and development of GC (15, 86–88). In different microhabitats, it was observed that BDCA2^+^ pDCs and Foxp3^+^ Tregs were significantly increased in tumoral and peritumoral tissues, and there was a positive correlation between them (15). Moreover, pDCs can effectively promote the differentiation of naive CD4^+^ T cells into Tregs, thereby facilitating tumor immune escape (89). Interestingly, TLR agonist stimulation caused metabolic reprogramming in DCs, which was critical for immune activation (59). Basit et al. demonstrated that TLR-stimulation of pDCs significantly increases the expression level of genes that regulate oxidative phosphorylation and glutamine metabolism, thereby promoting pDC activation, leading to higher production of IFN-α and inducing T cell responses (90).

### PDCS and Breast Cancer

Breast cancer (BC) is the most frequent malignancy and the second biggest cause of cancer- associated mortality in women worldwide (91, 92), and approximately 70-80% of patients with early stage and non-metastatic disease can be cured (93). The most aggressive type of breast cancer is triple-negative breast cancer (TNBC), which does not express of HER2/neu, estrogen receptor and progesterone receptor (91). In a large majority of cases, immunity against breast cancer does not exhibit a protective effect, which indicates that breast cancer cells escape immunosurveillance (91). In addition, previous research has shown that the breast tumor microenvironment makes immune cells dysfunctional and is conducive to immunosuppression, thereby preventing the establishment of anti-tumor immunity (94). Published studies have shown that pDCs infiltrate breast tumors, but are impaired by TGF-β and TNF-α to produce IFN-α (95), and associated with poor clinical prognosis (64, 96), indicating that pDCs might contribute to the immune escape of breast tumors and ultimately promote their growth (96). Another study showed that the production of GM-CSF and pDCs infiltration was significantly increased in breast cancer, and pDCs activated by GM-CSF promoted the differentiation of CD4^+^ T cells into Tregs, leading to immunosuppression (64, 97, 98). In addition, pDCs-derived TNF-α in breast tumors triggered activation of the NF-κB signaling pathway in cancer cells, which in turn upregulated the expression level of CXCR4 and led to increased metastasis to lymph nodes, which ultimately promoted cancer progression (99, 100).

### PDCS and Liver Cancer

Liver cancer is the fifth most common malignancy in men and the ninth most commonly occurring cancer in women, which can be divided into primary liver cancer and secondary or metastatic liver cancer according to its cause, and has a poor prognosis. The therapeutic effect of chemotherapy in liver cancer is very limited, and it can only prolong the survival of patients by 2.3 to 2.8 months on average (101). The immune regulation in the liver tumor microenvironment may contribute to the immune escape of tumor cells, thereby greatly reducing the efficacy of immunotherapy (102). Previous studies have shown that pDCs also heavily infiltrate liver cancer tissues, which promotes vascular invasion and lymph node metastasis, resulting in a shorter overall survival and a higher recurrence rate for patients (103). Like melanoma (39), pDCs exposed to liver tumor-derived factors increased the expression levels of ICOSL to promote Tregs to produce increased IL-10, thereby strongly inhibiting T cell responses and ultimately assisting immunosuppression and tumor progression (12). Furthermore, the increase of intratumoral pDCs was associated with increased infiltration of Tregs, therefore, the evaluation of intratumoral pDCs represents an excellent predictor of the prognosis of liver cancer patients (103).

### PDCS and Squamous Cell Carcinoma

Squamous cell carcinoma (SSC) is a tumor of the upper aerodigestive tract with high fatality rate and poor prognosis (104, 105). In primary oral squamous cell carcinoma (OSCC), tumor cells produce high levels of CXCL12 (106), which promotes the infiltration of pDCs expressing the corresponding receptor CXCR4, which has also been observed in head and neck SSC (107). However, there is evidence that tumor-induced down-regulation of TLR9 in pDCs was observed within the tumor environment (108), and simultaneously various cytokines in the tumor microenvironment such as VEGF, TGF-β and IL-10 inhibit the maturation and activation of TIpDCs (109), resulting in a significant decrease in the expression of IFN-α, indicating TIpDCs dysfunction (108, 110). In addition, the increase in the number of TIpDCs is associated with lymph node metastasis and overall survival (110). Another study showed that the use of CD317 antibody to deplete pDCs in the tumor microenvironment significantly promoted the recovery of T cell function, and inhibited the tumor infiltration of Tregs and monocyte-derived suppressor cells, thereby breaking the immunosuppressive state (60). This further supports that the high infiltration of pDCs in tumors promotes the progression of SSC.

The subtyping of pDCs is also of great significance in SSC. BDCA2 is a specific marker of human pDCs, but high BDCA2 is expressed by immature pDCs, while pDCs expressing CD123 have higher maturity and ability to secrete cytokines (60, 111). In head and neck SSC, the Poropatich group identified a subgroup pDCs expressing high levels of OX40 in the tumor microenvironment, which is conducive to anti-tumor immunity by increasing the expression levels of local IL-12 and IFN-α and enhancing the interaction between cDC and CD8^+^ T cell *via* OX40/OX40L-signaling axis (112). Additionally, CD56^+^ pDCs express higher levels of perforin and granzyme b, which confers strong cytotoxic activity, but the proportion of such cells is significantly decreased in head and neck SSC (113). Similar studies have shown that pDCs can be divided into two subgroups through the expression level of CD2, where CD2^high^ pDCs secrete higher levels of IL12p40 and express higher levels of costimulatory molecule CD80, and exhibit higher efficiency in triggering T cell proliferation (114). It can be seen that up-regulating CD56 or CD2 of pDCs will have a positive effect on anti-tumor immunity in SSC. Therefore, whether the combination of chemoradiation and intratumor injection of activated pDCs could also improve clinical outcome in patients is worthy of further study.

### PDCS and Leukemia

Leukemia is the common name for several malignant disorders, which are manifested by a robust increase in the number of leucocytes in the blood and/or the bone marrow (115). Previous studies have found that infiltrating CD123^+^ pDCs have been observed in the hematopoietic tissues of a fraction of chronic myelomonocytic leukemia (CMML) patients, and the excess of pDCs is associated with the accumulation of Tregs and the increased risk of acute leukemia transformation (33). However, in chronic myeloid leukemia (CML), pDCs are derived from precursors that express a low level of *BCR*-*ABL*, and develop normally and usually express the co-stimulatory antigen CD86. In addition, CML-pDCs also retain their ability to mature and produce IFN-α, thereby regulating anti-leukemic immunity in CML (116). On the other hand, due to different clinical and pathological manifestations, pDC neoplasms can be divided into two types including mature pDC proliferation associated with myeloid neoplasms and blastic pDC neoplasm (BPDCN) (117), and BPDCN is an aggressive hematopoietic clonal neoplasm that prone to leukemia transformation and poor prognosis (118). And in acute myeloid leukemia (AML), Zalmai et al. identified a group of pDC-AML with completely different phenotype from BPDCN, with high expression of CD34 and CD303, low expression of CD123 and cTCL1, and no expression of CD56 (119). Molecular analysis indicated that these pDCs were inactive and neoplastic, and exhibited frequent RUNX1 mutations (119). Moreover, studies showed that clinical use of tagraxofusp (SL-401) completely inhibited protein synthesis leading to cell death of pDCs, which had a positive effect on inhibiting acute transformation in leukemia (61, 118).

### PDCS and Ovarian Cancer

Ovarian cancer (OC) is the most aggressive gynecological cancer in women (11). High infiltration of pDCs is significantly associated with early relapse in ovarian cancer (11, 65, 120). At the same time, TApDCs not only exhibit less production of IFN-α, mainly mediated through tumor-derived TNF-α and TGF-β (120), but also induce tumor infiltration of ICOS^+^ Foxp3^+^ Tregs and drive immunosuppression *via* ICOS/ICOSL stimulation (65). Additionally, both TApDCs and ICOS^+^ Foxp3^+^ Tregs predict disease progression in epithelial ovarian cancer patients (65). On the other hand, published reports indicate that pDCs control the homeostasis of CD4^+^ Foxp3^+^ Tregs and Th17 cells *in vivo* by expressing sialic acid-binding Ig-like lectin (Siglec)-H (121). And pDCs in tumor ascites induced IL-10^+^ CCR7^+^ CD45RO^+^ CD8^+^ Tregs which was independent of CD4^+^ CD25^+^ T cells, and inhibit tumor-associated antigen-specific T cell effector functions through IL-10 (122). Moreover, the results of Zou et al. showed that high expression of CXCL12 was observed in malignant human ovarian epithelial tumor cells, and CXCL12 induced adhesion, transmigration and chemotaxis of pDCs, and inhibited tumor macrophage IL-10-induced pDC apoptosis through CXCR4, resulting in poorly proliferating T cells (13). Interestingly, the Figdor group tested the immunomodulatory capacity of prophylactic live-attenuated and inactivated viral vaccines on pDCs, and found that prophylactic vaccines significantly induce the activation and maturation of pDCs, the expression of MHC class I and class II, and the production of IFN-α, that transform pDCs from an immunosuppressed state to an immune activated state (62). The above studies provide new ideas for the remission and treatment of ovarian cancer, by targeting ICOS/ICOSL to inhibit the accumulation of ICOS^+^ Foxp3^+^ Tregs in ovarian cancer, thereby eliminating immunosuppression. In addition, it can also play a positive role in restoring the function of pDCs by regulating the expression of Siglec-H. Using viruses as vaccine vectors to activate pDCs is also a new regulatory idea, but it should be noted that even live-attenuated viral, the impact of the virus itself must be considered.

## Conclusions

pDCs are an promising target for cancer immunotherapy; however, accumulating evidence indicates that the complex interaction of pDCs with tumor cells and their microenvironment appears to contribute to immunologic tolerance (7). In many types of cancers, tumor cells secrete the chemokine CXCL12 to induce the infiltration of a large number of immature pDCs, and the cytokines of VEGF, TNF-α, TGF-β and IL-10 secreted in tumor microenvironment inhibit the maturation and activation of pDCs, then make it unable to produce IFN-α. Additionally, pDCs recruit a large number of Tregs to the tumor site, leading to immunosuppression and promoting tumor growth. Therefore, it can provide ideas for clinical treatment of cancer in two aspects. On one hand, the injection of drugs to induce the activation of pDCs in tumors or the combination of chemoradiation and intratumor injection of activated pDCs may reverse the tumor microenvironment, thereby inhibiting tumor growth and improving patient survival rate (51, 105). On the other hand, we can eliminate the immunosuppressive effect in the tumor microenvironment by reducing the proportion of Tregs or inhibiting its function in the tumors. Published study demonstrated that daclizumab, a humanized anti-CD25 antibody, can block the IL-2 signaling pathway by binding to CD25, which in turn leads to the death of Tregs (123). In addition, some chemotherapeutic drugs can also reduce the number of Tregs by inhibiting Tregs gene synthesis and reducing cell expansion. Zhang et al. showed that the chemotherapeutic agent gemcitabine significantly reduced immunosuppression in the tumor microenvironment, accompanied by a decrease in Tregs and MDSCs, thereby inhibiting tumor growth (124). Furthermore, CTLA-4 is expressed on the surface of Tregs and transmits inhibitory signals in the immune response. Therefore, using monoclonal antibody to block the expression of CTLA-4, can reduce the inhibitory activity of Tregs, and achieve tumor suppression effects (125). However, more efforts are needed to develop more effective pDCs activators or Tregs inhibitors, which will help in the treatment of various malignancies clinically.

## Author Contributions

All authors listed have made direct and substantial contribution to this work, and approved the final manuscript.

## Funding

This work was supported by grant from National Natural Science Foundation of China (Grant No. 32000491), and also sponsored by projects from Henan Province to YL (No. 21IRTSTHN030 and GZS2021002).

## Conflict of Interest

The authors declare that the research was conducted in the absence of any commercial or financial relationships that could be construed as a potential conflict of interest.

## Publisher’s Note

All claims expressed in this article are solely those of the authors and do not necessarily represent those of their affiliated organizations, or those of the publisher, the editors and the reviewers. Any product that may be evaluated in this article, or claim that may be made by its manufacturer, is not guaranteed or endorsed by the publisher.
